# Etymologia: *Dracunculus medinensis*

**DOI:** 10.3201/eid2208.ET2208

**Published:** 2016-08

**Keywords:** etymologia, Dracunculus medinensis, Guinea worm, nematode, parasites, eradication, Africa

## *Dracunculus medinensis* [drə-kungʹku-ləs med-in-enʹsis]

Also known as Guinea worm (Figure) for its high prevalence along the Gulf of Guinea, *Dracunculus medinensis* (“little dragon from Medina”) is a parasitic nematode that infects humans and domestic animals through contaminated water. *D. medinensis* was described in Egypt as early as the 15th century bce and may have been the “fiery serpent” of the Israelites described in the Bible.

**Figure:**
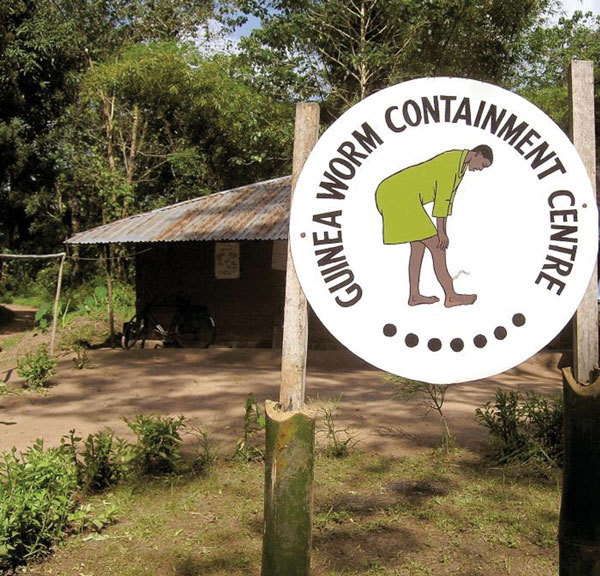
This 2004 photograph depicted the entrance to a Nigerian Guinea worm containment center. The sign at the entrance displayed a drawing of a Guinea worm sufferer. Photo by E. Staub, CDC/Carter Center.

Guinea worm disease was once a substantial cause of illness in tropical and subtropical Africa and Asia, but cases declined as water sanitation improved in the 19th century. In 1986, the World Health Organization resolved to eradicate the parasite, and in 2015, there were only 22 cases in 4 countries (Chad, Ethiopia, Mali, and South Sudan).
